# Strawberry Decreases Intraluminal and Intestinal Wall Hydrolysis of Testosterone Undecanoate

**DOI:** 10.3390/molecules26010233

**Published:** 2021-01-05

**Authors:** Atheer Zgair, Yousaf Dawood, Suhaib M. Ibrahem, Jong Bong Lee, Wanshan Feng, Peter M. Fischer, Pavel Gershkovich

**Affiliations:** 1 College of Pharmacy, University of Anbar, Ramadi 31001, Iraq; ph.yousifdawood@uoanbar.edu.iq (Y.D.); ph.su82haib@uoanbar.edu.iq (S.M.I.); 2 School of Pharmacy, University of Nottingham, Nottingham NG7 2RD, UK; myjblee@gmail.com (J.B.L.); Wanshan.Feng@nottingham.ac.uk (W.F.); Peter.Fischer@nottingham.ac.uk (P.M.F.); Pavel.Gershkovich@nottingham.ac.uk (P.G.)

**Keywords:** testosterone undecanoate, FaSSIF, intestinal degradation, first-pass metabolism, strawberry

## Abstract

Male hypogonadism is often treated by testosterone (T) replacement therapy such as oral administration of the ester prodrug, testosterone undecanoate (TU). However, the systemic exposure to T following oral TU is very low due to esterase-mediated metabolism, particularly in the small intestine. The aim of this work was to examine the esterase-inhibitory effect of natural fruit extract of strawberry (STW) on the intestinal degradation of TU as a potential approach to increasing the oral bioavailability of T. Herein, the hydrolysis of TU was assessed in fasted state simulated intestinal fluid with added esterase activity (FaSSIF/ES) and Caco-2 cell homogenates in the presence of STW extract. It is noteworthy that STW substantially inhibited the degradation of TU in FaSSIF/ES and Caco-2 cell homogenates at concentrations that could be achieved following oral consumption of less than one serving of STW fruit. This can significantly increase the fraction of unhydrolyzed TU in the intestinal lumen as well as in enterocytes. In addition, it was demonstrated that TU has high intestinal lymphatic transport potential as the association of TU with plasma-derived human chylomicrons was in the range of 84%. Therefore, oral co-administration of TU with STW could potentially increase the intestinal stability of TU and consequently the contribution of lymphatically delivered TU to the systemic exposure of T in vivo.

## 1. Introduction

**Figure 1:**
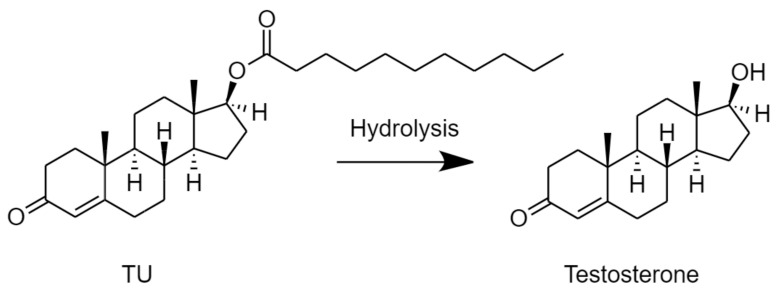
Hydrolysis of testosterone undecanoate (TU) to testosterone.

Testosterone (T) is an essential androgen in the development of the male reproductive system and sexual characteristics [1]. Inadequate release of testicular T is associated with male hypogonadism, a clinical condition that affects the quality of life [2]. Symptomatic hypogonadism is usually treated by T replacement therapy [3]. T is available as a transdermal patch and gel, subdermal implants, buccal tablet, and nasal gel [3]. T is well absorbed from the gastrointestinal tract. However, the extensive first-pass metabolism of T limits its use as an orally effective therapeutic option in the treatment of hypogonadal disorders [4]. The ester prodrug of T, testosterone undecanoate (TU), was approved as an orally effective androgen [5]. TU is metabolised by non-specific plasma esterases to T (Figure 1) [6]. It was demonstrated that TU increases systemic T exposure predominantly by enhancing intestinal lymphatic transport, thus avoiding first-pass metabolism in the liver [7,8,9], yet, the systemic exposure to T following oral administration of TU was less than 7% in humans [10]. Several human and animal studies have revealed that a large fraction of TU is metabolised in the intestinal lumen and intestinal wall. The metabolites, primarily T, are absorbed via the portal vein to the liver where they are further extensively metabolised. Moreover, the small fraction of TU that bypasses the intestine is metabolised in the liver [7,8,9,11]. The uptake of TU by chylomicrons (CM) in enterocytes during the process of intestinal lymphatic transport was proposed as the primary pathway for the bioavailable T. This pathway could reduce the fraction of TU being hydrolysed in the wall of the intestines as well as in the liver [7,12]. We have recently suggested that decreasing the intestinal metabolism of TU could be a valid approach to enhancing the oral bioavailability of T as the intestinal wall, as opposed to the liver, is the site where around 90% of the absorbed TU is metabolised [12].

In addition, it has been demonstrated that fruit extracts, particularly strawberry (STW), can significantly inhibit the intestinal degradation of the esterase-sensitive antiviral prodrug tenofovir disoproxil [13,14]. Therefore, the present study was designed to evaluate the inhibitory effect of STW on the intestinal metabolism of TU as a strategy to increase the availability of TU for intestinal lymphatic transport and hence the systemic exposure to T.

## 2. Materials and Methods

### 2.1. Materials

TU and Vitamin D_3_ were purchased from Beijing Sjar Technology Development (Beijing, China) and Alfa Aesar (Lancashire, UK), respectively. Hank’s balanced salt solution (HBSS), 4-2-hydroxyethyl-1-piperazineethanesulfonic acid (HEPES) buffer, lecithin, porcine liver esterase, FBS, sodium taurocholate (NaTc), NaH_2_PO_4_, NaCl, KBr, fast blue BB salt (4-benzoylamino-2,5-dimethoxybenzenediazonium chloride hemi (zinc chloride) salt), and gallic acid were obtained from Sigma (Gillingham, UK). Dulbecco’s modified Eagle medium (DMEM) with added GlutaMAX™, D-glucose, and HEPES was procured from Gibco (Paisley, UK).

### 2.2. Determination of Total Phenolics in STW

Phenolic compounds are important components of STW that have demonstrated biological activities [15]. In this study, total phenolic contents were determined to estimate the amount of fresh STW fruit that is equivalent to the amount of freeze-dried STW powder. Phenolic contents (gallic acid equivalents) in freeze-dried STW powder and fresh STW fruits were determined by fast blue BB assay according to the literature [16]. Briefly, freeze-dried powder (0.25 g) and pieces of freshly cut STW fruit (2.5 g) were homogenised with 3 mL and 12 mL of 70% aqueous methanol (*v*/*v*), respectively, at 12,000 rpm for 30 s (POLYTRON^®^, Luzern, Switzerland). Samples were then subjected to ultrasonication for 30 s followed by centrifugation (6650× *g*, 10 min, room temperature). Supernatants were collected in new tubes and diluted in water (1:20; *v*/*v*). One-hundred and fifty microliter of diluted samples, gallic acid standards, or water was placed in a flat and clear bottom 96-well plates. Fast blue BB solution (1%, 15 µL) was added and mixed for 30 s. Next, an NaOH solution (1 M, 15 µL) was added and mixed for 30 s. Samples were incubated for 90 min at room temperature, protected from light. Absorbance was then measured at 420 nm (EnVision^®^ Multilabel Plate Reader, PerkinElmer Inc., Waltham, MA, USA). Total phenolic content was then determined as gallic acid equivalent based on a calibration cure of gallic acid standards.

### 2.3. Stability of TU in Fasted State Simulated Intestinal Fluid with Added Esterase Activity (FaSSIF/ES)

The stability of TU was assessed in fasted state simulated intestinal fluid with added esterase activity (FaSSIF/ES; 20 IU/mL) in the presence of 0%, 2%, and 4% STW extract (weight of freeze-dried STW powder per volume of the medium). FaSSIF was prepared according to the literature [17]. The stability of TU in FaSSIF/ES was performed as described in the literature [18,19]. The reaction medium (1 mL) was incubated at 37 °C for 5 min prior to the experiment. The reaction was then initiated by the addition of TU to yield a concentration of 10 μM. Samples (100 µL each) were withdrawn at 0, 15, 30, 45, and 60 min. Three-hundred microliter of ice-cold acetonitrile (ACN) was immediately added to the samples to stop the reaction. Later, samples were prepared for HPLC-UV analysis in the same day as described below. In addition, TU stability was assessed in FaSSIF without esterase to determine the chemical stability. The depletion rate constant of TU (*k*) was obtained from the plot of logarithmic concentrations of TU (% of concentration at time 0) versus time. The elimination half-life (*t*_1/2_) of TU depletion was then calculated according to Equation (1). Experiments were performed in quadruplet.

*t*_1/2_ = 0.693/*k* (1)

### 2.4. Stability of TU in Caco-2 Cell Homogenates

The stability of TU was assessed in human intestinal Caco-2 cell homogenates in the presence of 0%, 2%, and 4% STW extract. For ester prodrug stability studies, Caco-2 cell homogenate was demonstrated as an equivalent alternative to human intestinal homogenate [20] and has been applied to predict the first-pass metabolism of TU in the small intestine [12]. The preparation of Caco-2 cell homogenate was described before [12]. Briefly, cells were seeded in culture flasks at a density of 3.75 × 10^4^ cells/cm^2^. Cells were maintained at 37 °C, 95% relative humidity, and 5% CO_2_ for 21 days. Then, confluent cells were scraped and lysed by ultrasonication followed by centrifugation (10,000× *g*, 5 min, 4 °C) to obtain the supernatant. The concentration of proteins was adjusted to 1 mg/mL for the stability study.

The stability of TU in Caco-2 cell homogenate was assessed as described before [12]. Briefly, cell homogenates (1 mL) were spiked with TU to obtain a concentration of 10 μM. Homogenates were then incubated for 1 h at 37 °C with continuous shaking at 200 rpm. Samples (100 µL each) were withdrawn at 0, 15, 30, 45, and 60 min. Three-hundred microliter of ice-cold ACN was immediately added to the sample to stop the hydrolysis. Later, samples were prepared for HPLC-UV analysis in the same day as described below. Experiments were performed in quadruplet.

### 2.5. Assessment of Intestinal Lymphatic Transport Potential

The intestinal lymphatic transport potential of TU was evaluated by determining the association of TU with plasma-derived human CM as previously described [21]. To note, CM association is a key step in the intestinal lymphatic transport of drugs [21,22]. The ethical approval for this experiment was obtained from the Faculty of Medicine and Health Sciences Research Ethics Committee, Queens Medical Centre, Nottingham University Hospitals (BT12102015 CBS SoP). Three healthy human males (25–40 years old) that had not been on any medication one-week prior experiment were enrolled in this study. Following overnight fasting, participants received a high-fat breakfast (full English breakfast or olive oil-fried eggs). Thirty millilitres of blood samples were collected in heparinised tubes 3–4 h after breakfast. Plasma was separated by centrifugation (800× *g*, 10 min, 15 °C). CM were harvested from plasma using a density gradient technique [21]. Briefly, the density of plasma aliquots was adjusted to 1.1 g/mL by KBr. Four millilitres of plasma were placed in polyallomer ultracentrifuge tube and standard solutions of PBP with densities of 1.063, 1.019, and 1.006 g/mL were layered on top. Following ultracentrifugation (268,350× *g*, 35 min, 15 °C; SORVALL^®^ TH-641 Rotor, Thermo Fisher Scientific, Gloucester, UK), the top 1 mL layer containing CM was carefully decanted. Triglyceride concertation in CM emulsion was determined by triglycerides enzymatic kit according to the manufacturer protocol (Sigma Aldrich, Dorset, UK) and adjusted to 100 mg/dL by dilution with standard PBS solution (1.006 g/mL density). Pooled CM emulsion from all volunteers was stored in the fridge at 4–8 °C until the uptake experiments (<24 h).

The uptake of TU by human CM emulsion was performed as described in the literature [22]. TU was spiked to 1 mL of CM emulsion in a test tube to achieve molar concentration of 1.75 μM. Reaction tubes were incubated for 1 h at 37 °C with continuous mixing. Following incubation, 900 μL of CM emulsion was transferred to a new tube and the density was adjusted to 1.1 g/mL using KBr. CM were separated by ultracentrifugation (268,350× *g*, 35 min, 15 °C; SORVALL^®^ TH-641 Rotor, Thermo Fisher Scientific, UK). Following ultracentrifugation, the upper 1 mL layer was carefully decanted and processed for HPLC-UV analysis on the same day, as described below. The concentration of TU was also determined in CM emulsion prior to density gradient ultracentrifugation. The association of TU with human CM was calculated according to Equation (2).

(2) $$\mathrm{Association}\;\mathrm{of}\;\mathrm{TU}\;\mathrm{with}\;\mathrm{human}\;\mathrm{CM}\;\left(\%\right)=\;\frac{\mathrm{Amount}\;\mathrm{of}\;\mathrm{TU}\;\mathrm{in}\;\mathrm{CM}\;\mathrm{emulsion}\;\mathrm{following}\;\mathrm{ultracentrifugation}\;}{\mathrm{Amount}\;\mathrm{of}\;\mathrm{TU}\;\mathrm{in}\;\mathrm{CM}\;\mathrm{emulsion}\;\mathrm{at}\;\mathrm{the}\;\mathrm{end}\;\mathrm{of}\;\mathrm{incubation}}\times \;100$$

### 2.6. Analytical Methods

The determination of TU concentrations in FaSSIF, Caco-2 cell homogenates, and CM emulsion was performed using Waters Alliance HPLC-UV system (Waters Corporation, Milford, MA, USA) as previously described [12]. Concisely, 300 µL of ACN and 20 µL of the internal standard (vit D_3_, 50 µm) were added to 100 µL of sample and vortexed for 1 min. Two-hundred microlitre of HPLC-grade water was added and vortexed for 1 min. Three millilitres of n-Hexane were added followed by 10 min vortex-mixing for extraction. Samples were then centrifuged (1160× *g*, 10 min, 15 °C) and the upper organic layer was transferred to a new tube and evaporated under N_2_ stream. Samples were redissolved in 100 µL ACN and 20 µL was injected for HPLC-UV analysis.

Analytes were separated on ACE C18 (4.6 mm ID × 10 cm) column at 50 °C (Hichrom Ltd., Reading, UK). The mobile phase was an isocratic mixture of ACN and water (96:04; *v/v*). The flow rate was set at 0.5 mL/min and the absorbance was monitored at 240 nm for 20 min. Empower^TM^ 2 software was used for data processing. The elution time of TU was 11.3 min. For FaSSIF and Caco-2 cell homogenate samples, analyte/internal standard peak area ratios were determined and normalised to the value at time zero. For CM emulsion samples, calibration curves were constructed and used to calculate TU concentration. The analytical method was validated for selectivity, accuracy, and precision in accordance with the FDA Guidance for Bioanalytical Method Validation [23]. The lower limit of quantification was 0.2 µM. Inter- and intra-day precision and accuracy were below 15% RSD and RE, respectively.

### 2.7. Statistical Analysis

The results are expressed as mean ± standard deviation (SD). The statistical significance between data sets was analysed using ANOVA with Dunnett’s multiple comparisons test. A *p* < 0.05 was considered statistically significant.

## 3. Results

### 3.1. Phenolic Contents in Freeze-Dried STW and Fresh STW Fruit

The determination of phenolic contents has demonstrated that each 10 g of freeze-dried STW powder is equivalent to approximately 90 g of fresh STW fruit. Total phenolics in freeze-dried STW powder were 0.67 ± 0.09 g (gallic acid equivalent in 10 g freeze-dried powder, *n* = 3). In fresh STW fruit, total phenolics were 0.76 ± 0.12 g (gallic acid equivalent in 100 g fresh STW, *n* = 5).

### 3.2. Stability of TU in FaSSIF/ES

**Figure 2:**
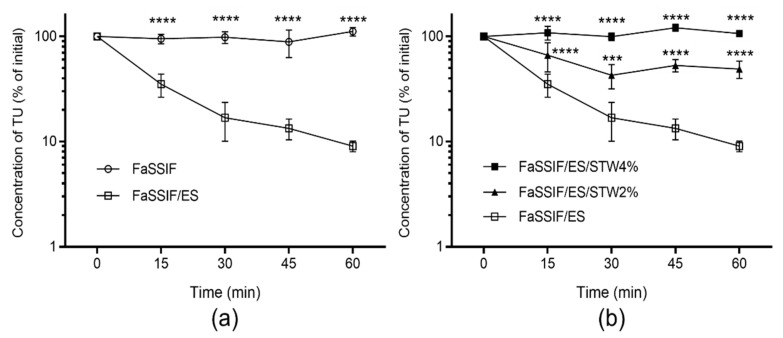
Stability of testosterone undecanoate (TU) in fasted state simulated intestinal fluid (FaSSIF) with esterase (ES) activity (20 IU/mL) and without ES (**a**) and in the presence of 0%, 2%, and 4% strawberry (STW) extract (**b**). Values are presented as a percentage of TU concentration at time 0 (mean ± SD, *n* = 4). Two-way ANOVA with Dunnett’s multiple comparisons test was used for statistical analysis. Statistical differences compared to FaSSIF/ES group; *** *p* < 0.001; **** *p* < 0.0001.

The stability of TU was tested in FaSSIF/ES to simulate physiological conditions in the small intestine. The depletion of TU in the reaction medium corresponded to the hydrolysis of TU by esterases. The hydrolysis of TU in FaSSIF/ES is shown in Figure 2a, which also shows that TU is stable in simulated intestinal fluid without added esterase, indicating chemical stability in FaSSIF over the incubation period (60 min). As shown in Figure 2b, the hydrolysis of TU in the presence of esterase was substantially inhibited when co-incubated with 2% and 4% STW extracts in a dose-dependent manner. The *t*_1/2_ depletion of TU in FaSSIF/ES was 18.9 ± 1.6 min, which was increased 13-fold in the presence of 2% STW extract. In the presence of 4% STW extract, TU was stable over the reaction time, and therefore *t*_1/2_ depletion could not be calculated.

### 3.3. Stability of TU in Caco-2 Cell Homogenate

**Figure 3:**
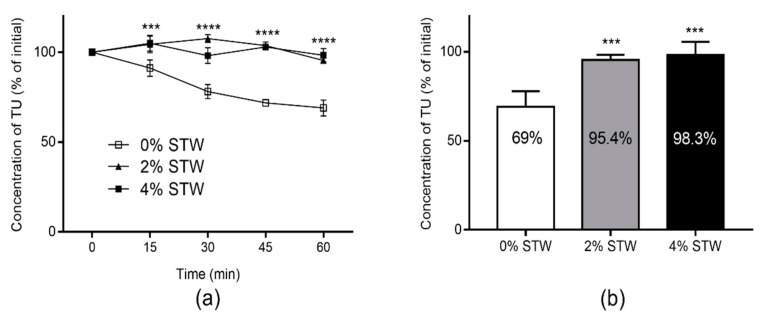
(**a**) The hydrolysis of testosterone undecanoate (TU) in Caco-2 cell homogenates in the presence of 0%, 2%, and 4% strawberry (STW) extract; (**b**) The unhydrolyzed fraction of TU dose following 60 min incubation. Values are expressed as percentage of TU concentration at time 0 (mean ± SD, *n* = 4). Statistical analysis was performed using one-way and two-way ANOVA with Dunnett’s multiple comparisons test, as appropriate. Statistical differences of 2% and 4% STW groups were similar when compared to STW-free Caco-2 cell homogenate group; *** *p* < 0.001; **** *p* < 0.0001.

The stability of TU was tested in Caco-2 cell homogenate to assess the effect of STW on the metabolic activity in the intestinal wall. The hydrolysis-time profiles of TU in Caco-2 cell homogenates in the presence of different concentrations of STW extract are shown in Figure 3a. STW has significantly inhibited the hydrolysis of TU in Caco-2 cell homogenates at the tested concentrations (2% and 4%). The unhydrolyzed fractions of TU dose at the end of incubation are presented in Figure 3b.

### 3.4. Intestinal Lymphatic Transport Potential of TU

Affinity to CM is a prerequisite for efficient intestinal lymphatic transport [21,24], which had earlier been demonstrated to be linearly proportional to the value of CM association [22]. In this study, TU showed high association values with natural human CM (84.3 ± 19.8%, mean ± SD, *n* = 5).

## 4. Discussion

T replacement therapy is often used to treat symptomatic hypogonadism in men. Therapeutic levels of T can be achieved following oral administration of TU. However, the systemic exposure to T following oral TU is very low due to substantial first-pass metabolism, particularly in the small intestine [12]. Herein, we have evaluated the inhibitory effect of natural fruit extract of STW on the intestinal degradation of TU as a potential approach to increasing the oral bioavailability of T.

In general, following oral administration, pre-systemic degradation of a prodrug in the intestinal lumen could considerably decrease the fraction of dose available for systemic absorption. For ester prodrugs, intestinal degradation could be due to chemical and/or enzymatic hydrolysis [25]. Multiple hydrolysing enzymes are present in the lumen of intestine, of which carboxylesterases are highly expressed [26]. In the current study, TU was chemically stable over 60 min of incubation in physiologically simulated intestinal fluid, FaSSIF (Figure 2a). However, the addition of esterase activity, which corresponds to a mixture of esterases that include carboxylesterase, resulted in the rapid hydrolysis of TU in FaSSIF with a depletion *t*_1/2_ of 18.9 min. Consequently, more than 90% of TU was degraded within 60 min (Figure 2a). Therefore, it is estimated that a large proportion of the administered dose could be degraded in the intestinal lumen. This can markedly decrease the fraction of TU absorbed into the intestinal wall, thus reducing the bioavailability of T following oral administration of TU in vivo.

In the presence of 2% freeze-dried STW, the hydrolysis of TU in FaSSIF/ES was substantially decreased, preserving more than 50% of TU from being hydrolysed by esterases over the incubation period (Figure 2b). In addition, TU was almost completely preserved from hydrolysis when co-incubated with 4% freeze-dried STW (Figure 2b). Therefore, STW could significantly increase the fraction of TU available for absorption in the intestinal lumen. As TU is a highly lipophilic molecule, the fraction available for absorption can also be limited by the low solubility in the intestinal lumen. Thus, in addition to esterase inhibition, formulation approaches could be applied to increase the fraction of TU available for absorption into the intestinal lumen.

Experimental determination of phenolic content has demonstrated that oral intake of 45 g and 90 g of fresh STW fruit can produce intestinal concentrations equivalent to 2% and 4% of freeze-dried STW, respectively, assuming intestinal fluid volume of 250 mL. To note, it is estimated that one serving of STW fruit is around 140 g [27]. Therefore, achieving esterase-inhibitory concentrations in the intestinal lumen is highly likely following the consumption of less than one serving of STW fruit. The esterase-inhibitory effect of STW could be attributed to the fact that STW is rich in multiple ester compounds, which could competitively inhibit the hydrolysis of ester prodrugs [14].

Further, we used Caco-2 cell homogenates to assess the effect of STW on the intestinal first-pass metabolism of TU. In fact, we previously applied the degradation of TU in Caco-2 cell homogenate to predict the fraction of TU that could escape intestinal metabolism, which was as low as 11% of the absorbed fraction [12]. In this study, STW substantially altered the depletion profile of TU in Caco-2 cell homogenates (*p* < 0.001; Figure 3a). At 2% freeze-dried STW, the unhydrolyzed fraction of TU in Caco-2 cell homogenate increased to around 95% compared with 69% in STW-free homogenate over 60 min incubation (Figure 3b). This can potentially increase the fraction of TU that could survive metabolism in the intestinal wall in vivo. As the intestinal wall is the main site for first-pass metabolic loss of TU relative to the liver [12], it is anticipated that exposure to T could significantly be increased after oral administration of TU. The presence of 2% STW, as illustrated in Figure 3b, almost completely inhibited the degradation of TU, suggesting that ester compounds from STW saturated the hydrolysing enzymes. Therefore, upon increasing the concentration of STW to 4%, only a minor increase in the unhydrolyzed fraction of TU was observed.

As mentioned earlier, animal and human studies have shown that intestinal lymphatic transport of TU is the primary absorptive pathway for the majority of bioavailable T after oral administration of TU [7,8,9,11]. In this study, we assessed the intestinal lymphatic transport potential of TU by measuring the association with plasma-derived human CM. This approach was previously shown to provide accurate estimate for the intestinal lymphatic transport of lipophilic compounds [18,21,28]. The high association value of TU with CM in this study (84.3%) suggests considerable intestinal lymphatic transport and improved bioavailability following oral administration in conditions that promote CM assembly, particularly with long-chain triglyceride-rich lipids. The lipophilic drug 4,4′-dichlorodiphenyltrichloroethane (DDT) was reported to have comparable CM association value to TU (81.6%) [22]. Indeed, it was reported that around 22% of DDT dose was transported to the intestinal lymphatic system following oral administration of DDT with oleic acid in rats [29]. However, Coert et al. have shown that a low proportion of TU dose, approximately 2%, was recovered in the intestinal lymphatics of rats following oral administration with peanut oil [7]. Similarly, Shackleford et al. have demonstrated that around 3% of a TU dose was delivered to the systemic circulation through the intestinal lymphatics following oral administration of TU in dogs [8]. The authors suggested that the metabolic degradation in enterocytes could decrease the fraction of intact TU available for CM association. This is consistent with the metabolic profile of TU in Caco-2 cell homogenate and the low predicted fraction of TU that could escape intestinal metabolism as reported in our previous work (~11%) [12]. Therefore, the esterase-inhibitory effect of STW could retain more unhydrolyzed TU in the intestinal lumen and enterocytes. This means that more TU would be available for CM association and subsequent intestinal lymphatic transport, thereby augmenting systemic exposure to T.

## 5. Conclusions

In conclusion, it is prudent to suggest that oral administration of TU with less than one-serving of STW has the potential to decrease the hydrolysis of TU in the intestinal lumen, as well as in the intestinal wall. Coupled with the high CM association, increasing the unhydrolyzed fraction of TU in enterocytes could substantially increase the contribution of lymphatically-delivered TU to the systemic exposure of T. While the data of the current study are promising, further in vivo studies will be required to validate this approach as a strategy to enhance the bioavailability of T following oral administration of TU.

## Data Availability

Data contained within the article are available from the authors.
